# A Race Against Time: Endovascular Removal of an Intracardiac Foreign Body

**DOI:** 10.7759/cureus.106718

**Published:** 2026-04-09

**Authors:** Shamon Gumbs, Ifeoma Kwentoh, Eric S Atiku, Brian Donaldson

**Affiliations:** 1 Surgery, Harlem Hospital Center, Columbia University, New York, USA; 2 Internal Medicine, Harlem Hospital Center, Columbia University, New York, USA; 3 General Surgery, Harlem Hospital Center, Columbia University, New York, USA

**Keywords:** catheter embolization, central venous port complications, complications after port implantation, intracardiac foreign body, malrotation, thrombosis

## Abstract

Endovascular foreign body fragmentation, migration, and embolization to the right heart is a rare and late complication of the central venous port systems. The MediPort (Bard Medsystems, Reading, Massachusetts, USA) device consists of a port chamber attached to a central catheter implanted into the central venous system and used in patients with cancer for the administration of chemotherapy, parenteral nutrition, or blood transfusions. Interval radiologic survey, both intraoperatively and post-procedure, is crucial for investigating the possibility of both early and late complications, such as fracture and migration of the catheter, and for planning intervention. We detail a case of a 51-year-old woman with a background of estrogen receptor-positive invasive ductal carcinoma of the left breast, status post lumpectomy, neoadjuvant chemotherapy via right subclavian vein MediPort, and beam radiation. She presented for the removal of the MediPort device following completion of chemotherapy. However, intraoperatively, the catheter was noted to be 9 cm shorter in length, with an irregular tip, appearing incomplete from the original length, indicating fragmentation. Chest X-ray demonstrated a fractured catheter approximately 9 cm in length coursing from the right atrium to the right ventricle from its original tip at the distal superior vena cava. Vascular surgery emergently completed intracardiac foreign body retrieval from the right heart using the Bard 30 mm loop snare catheter system successfully.

## Introduction

Central venous port systems (CVPS) have been widely used for over three decades to provide reliable long-term venous access, particularly in oncology patients requiring chemotherapy, parenteral nutrition, or repeated blood transfusions [1]. These fully implantable devices significantly enhance patient's quality of life and demonstrate substantially lower infection rates compared to non-implantable central venous devices [2]. Despite their considerable benefits, CVPS are associated with various early and late complications, including infection (1-7%), thrombosis (1-5%), pneumothorax, cardiac arrhythmias, catheter malposition, and mechanical complications such as catheter pinch-off syndrome and fracture [3,4].

Catheter pinch-off syndrome represents a mechanical complication wherein the catheter undergoes chronic intermittent compression between the clavicle and the first rib, particularly when inserted via the subclavian approach medial to the mid-clavicular line [5]. This repetitive compression leads to progressive catheter deformation, thinning, and eventual transection. The syndrome typically manifests with difficulty infusing or aspirating through the port, positional flow variations, or complete catheter occlusion [6]. If unrecognized, pinch-off syndrome can progress to complete catheter fracture and intravascular embolization.

Although catheter fracture and embolization are rare complications, occurring in less than 0.1% of insertions, they pose significant risks, including life-threatening cardiac arrhythmias, cardiac perforation, pulmonary embolism, septic embolization, and right ventricular outflow tract obstruction [7,8]. Risk factors for catheter fracture include subclavian vein insertion (particularly medial approach), prolonged catheter dwell time (>12 months), repetitive mechanical stress, and certain catheter materials [9,10]. Recent evidence suggests that right-sided subclavian insertions carry a higher fracture risk than left-sided or jugular approaches [11].

The clinical presentation of catheter embolization varies widely, ranging from completely asymptomatic discovery during routine imaging to non-specific symptoms such as shoulder pain, neck discomfort, or palpitations, and in severe cases, chest pain, dyspnea, cardiac arrhythmias, or sudden cardiac arrest [12]. Notably, more than 70% of patients with embolized catheter fragments remain asymptomatic, making radiologic surveillance essential for early detection [13].

When catheter fracture and embolization occur, endovascular retrieval represents the gold standard of care, with reported success rates exceeding 90-95% [14,15]. Percutaneous retrieval techniques using loop snares, basket devices, or balloon catheters under fluoroscopic guidance offer minimal invasiveness with low complication rates compared with surgical extraction. However, prompt recognition and intervention are crucial to prevent migration to the distal pulmonary vasculature or cardiac complications.

Preventive strategies include routine pre-removal chest radiography to assess catheter integrity, preferential use of jugular or left subclavian approaches, lateral subclavian insertion technique, and heightened clinical suspicion in patients with long-term catheters or symptoms suggestive of catheter dysfunction. Despite these measures, catheter fracture remains an unpredictable complication requiring immediate recognition and management.

We present a case of a fractured MediPort (Bard Medsystems, Reading, Massachusetts, USA) catheter segment that migrated into the right heart chambers, necessitating urgent dual-access endovascular retrieval, and discuss the clinical implications, diagnostic approach, and management strategies for this rare but potentially catastrophic complication.

## Case presentation

**Figure 1:**
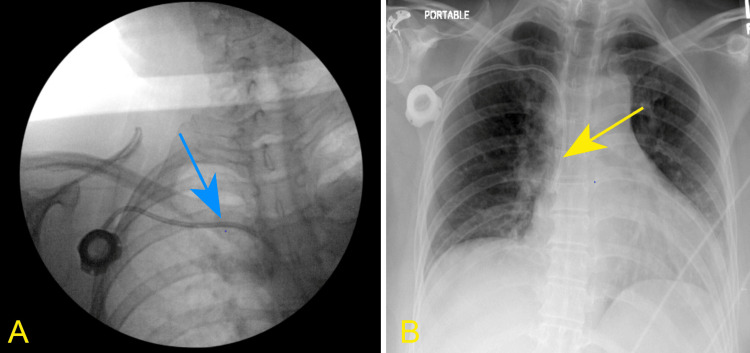
(A) Fluoroscopic placement of the intracardiac MediPort (blue arrow). (B) Chest X-ray demonstrating well-placed intracardiac tip of the MediPort (yellow arrow).

A 51-year-old woman with a history of estrogen receptor-positive invasive ductal carcinoma, grade 3, of the upper outer quadrant of the left breast (BI-RADS 6, biopsy-proven, diagnosed in July 2019, and MediPort placed in September 2019) presented electively for MediPort removal after the completion of chemotherapy (Figure 1). 

At presentation for elective port removal, the patient was completely asymptomatic with no complaints of chest pain, palpitations, dyspnea, shoulder discomfort, or positional symptoms during port access. She had completed her chemotherapy regimen six months before presentation and had no further need for the port. Physical examination revealed a well-healed port site without signs of infection, inflammation, or skin erosion. Vital signs were stable, and cardiovascular examination was unremarkable.

During the MediPort removal procedure under local anesthesia, the port chamber was successfully dissected free from the subcutaneous pocket. However, upon gentle traction on the catheter, only approximately 5 cm of the catheter was retrieved, significantly shorter than the expected length. The distal end of the retrieved catheter segment appeared irregular, frayed, and incomplete, with visible signs of mechanical transection rather than a smooth, intact catheter tip. This finding raised immediate concern for a fractured catheter and the retention of the distal fragment.

**Figure 2:**
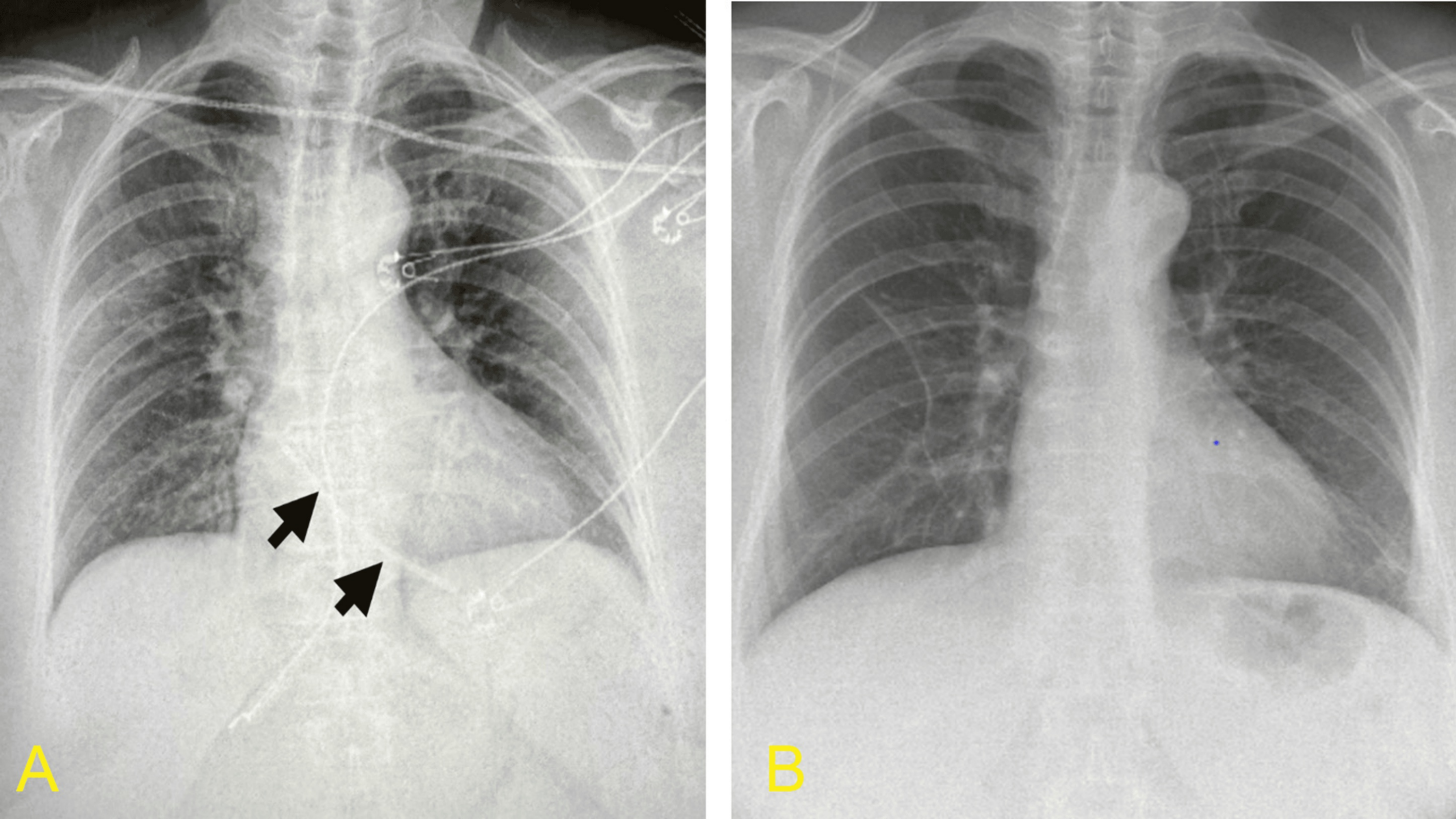
(A) CXR demonstrating the migrated and fragmented catheter segment lodged within the right atrium (black arrowheads). (B) Post-retrieval chest radiograph confirming complete removal of the intracardiac foreign body. CXR, chest X-ray.

An immediate intraoperative chest X-ray (CXR) was obtained, which revealed a radiopaque linear foreign body approximately 9 cm in length extending from the right atrium into the right ventricle (Figure 2A). The fractured catheter segment appeared to be free-floating within the right heart chambers. Given the significant risk of cardiac arrhythmias, right ventricular outflow tract obstruction, pulmonary embolization, and potential cardiac perforation, vascular surgery was urgently consulted.

The patient was started on therapeutic heparin to prevent thrombus formation on the foreign body surface and reduce the risk of thromboembolic complications. She was taken emergently to the operating room for endovascular foreign body extraction under fluoroscopic guidance.

Initial attempts to retrieve the catheter fragment were made via a right common femoral vein approach. After obtaining venous access with an 8-French sheath, a 7-mm gooseneck snare catheter was advanced under fluoroscopy through the inferior vena cava into the right atrium and right ventricle. Multiple attempts were made to capture the distal end of the catheter fragment; however, the fragment was highly mobile within the cardiac chambers, and the angle of approach from the femoral vein made secure capture challenging. The catheter fragment repeatedly slipped from the snare loop due to its smooth surface and the cardiac motion.

Given the unsuccessful femoral approach, a dual-access strategy was employed. The right internal jugular vein was accessed under ultrasound guidance, and a second 8-French sheath was placed. A 7-mm gooseneck snare was then advanced through the superior vena cava into the right atrium and right ventricle. Coordinated manipulation of both snares from the superior and inferior approaches allowed for stabilization of the fragment. During manipulation, the catheter fragment was successfully dislodged from the right ventricle and repositioned more proximally into the right atrium, with its distal tip directed toward the superior vena cava, a more favorable position for retrieval.

With the fragment in a more accessible position, a final retrieval attempt was made from the right femoral approach using a larger 10-mm Bard gooseneck snare. The larger snare diameter provided better capture capability. The snare was carefully advanced and positioned around the distal end of the catheter fragment. With gentle traction and fluoroscopic confirmation, the fragment was successfully grasped and secured within the snare loop. The entire foreign body was then carefully withdrawn through the femoral vein sheath and extracted intact (Figure 2B). Fluoroscopy confirmed complete removal of the catheter fragment with no residual foreign material in the cardiac chambers or venous system.

Post-procedurally, the patient remained hemodynamically stable with continuous cardiac monitoring. A repeat CXR confirmed complete removal of the intracardiac foreign body with no evidence of pneumothorax, hemothorax, or vascular injury. The patient was monitored in the hospital for three days, during which she remained asymptomatic without arrhythmias, chest pain, or respiratory symptoms. She was discharged on postoperative day 3 with instructions for routine follow-up at the vascular surgery clinic. At her two-week and three-month follow-up appointments, she remained asymptomatic with normal physical examination and no evidence of complications related to the retrieval procedure.

## Discussion

CVPS have become indispensable tools in modern oncology and chronic care, providing safe, reliable, and long-term venous access with documented benefits, including reduced infection rates (compared with external catheters), improved patient comfort, and enhanced quality of life [1,2]. However, despite their widespread use and overall safety profile, CVPS are not without risks. Complications can be broadly categorized into early (occurring within 30 days of insertion) and late (occurring beyond 30 days) events [3]. Early complications include pneumothorax (0.5-1%), hemothorax, arterial puncture, air embolism, catheter malposition, and cardiac arrhythmias related to guidewire or catheter tip irritation of the cardiac conduction system [4]. Late complications encompass infection (1-7%), catheter-related thrombosis (1-5%), catheter occlusion, skin erosion, port migration, and mechanical complications such as catheter pinch-off syndrome, fracture, and embolization [5,6].

Catheter pinch-off syndrome is a well-recognized mechanical complication that occurs when the catheter is chronically compressed between the clavicle and the first rib, typically at the costoclavicular space [7]. This phenomenon is most commonly associated with subclavian vein catheter insertion, particularly when the puncture site is medial to the mid-clavicular line. The repetitive compression during shoulder movements and respiration leads to progressive catheter deformation, thinning, and eventual transection [8]. The natural history of pinch-off syndrome progresses through several stages: initial intermittent compression causing positional flow variations, progressive catheter deformation visible on imaging, complete or near-complete catheter occlusion, and ultimately catheter fracture with potential embolization [9]. Clinical signs that should raise suspicion for pinch-off syndrome include difficulty infusing or aspirating through the port, positional flow variations (improvement with arm abduction or shoulder elevation), resistance during flushing, and inability to obtain blood return despite successful infusion [10]. In addition to pinch-off syndrome, catheter fracture can result from other mechanisms, including material fatigue from long-term use (particularly beyond 12-18 months), manufacturing defects, excessive mechanical manipulation during access or removal, and chemical degradation from certain chemotherapeutic agents [11,12]. The incidence of catheter fracture and embolization remains low, reported at less than 0.1% of all CVPS insertions, but the potential for catastrophic outcomes mandates vigilant surveillance and prompt intervention [13].

The clinical presentation of intravascular catheter embolization is highly variable and often non-specific, contributing to potential delays in diagnosis. Studies indicate that 50-70% of patients with embolized catheter fragments are completely asymptomatic at the time of discovery, with the diagnosis made incidentally during routine imaging or at the time of attempted device removal [14,15]. When symptoms do occur, they may include non-specific complaints such as shoulder or neck pain, chest discomfort, palpitations, or a sensation of "something moving" in the chest. Severe presentations, though rare, can include life-threatening cardiac arrhythmias (ventricular tachycardia, ventricular fibrillation), chest pain mimicking acute coronary syndrome, dyspnea, cardiac tamponade from myocardial perforation, or sudden cardiac arrest [12,13]. In our case, the patient was entirely asymptomatic at presentation, and the catheter fracture was discovered only intraoperatively during attempted device removal. This highlights the critical importance of intraoperative vigilance and immediate imaging when catheter integrity is questioned. The finding of a shortened catheter with an irregular, incomplete tip should immediately prompt chest radiography to assess for retained catheter fragments. Diagnostic imaging plays a central role in both detecting catheter complications and guiding management. Standard posteroanterior and lateral chest radiographs are typically sufficient to identify catheter position, integrity, and any evidence of fracture or migration [15]. Fluoroscopy is invaluable during retrieval procedures, allowing real-time visualization of catheter fragment position, cardiac motion effects, and snare manipulation. In complex cases, CT with intravenous contrast may provide additional anatomic detail regarding fragment location, relationship to cardiac structures, and presence of associated thrombus [1].

Once a catheter fracture and intravascular embolization are identified, prompt retrieval is strongly recommended to prevent potential complications. While some historical reports have suggested conservative observation for small, asymptomatic fragments lodged in peripheral pulmonary vessels, current consensus strongly favors active retrieval for fragments located in central veins, cardiac chambers, or proximal pulmonary arteries due to the risks of arrhythmia, perforation, thromboembolism, and infection [14,15]. Endovascular retrieval represents the gold standard approach, with reported success rates of 90-95% and low complication rates (1-3%) [14]. Multiple percutaneous techniques and devices are available for foreign body retrieval, with selection based on fragment size, location, morphology, and operator experience. Commonly employed devices include gooseneck snares (most commonly used), available in various loop diameters (5-35 mm), which are effective for capturing tubular or elongated foreign bodies; basket retrieval devices, originally designed for urological stone extraction, which can be effective for irregularly shaped fragments or those with hooks or barbs; balloon catheter techniques, where a balloon catheter can be advanced distal to the fragment, inflated, and withdrawn to drag the fragment proximally for easier capture [13]; and forceps devices such as endovascular forceps or alligator graspers, which can be used for fragments with accessible ends or irregular surfaces [15].

Access site selection is guided by fragment location and anatomy. Femoral vein access is typically the first choice for fragments in the inferior vena cava, right atrium, or right ventricle, as it provides a relatively straight path and allows for larger sheath sizes. Internal jugular vein access is preferred for fragments in the superior vena cava or when a superior approach offers better angulation. In challenging cases, as demonstrated in our patient, a dual-access approach using both femoral and jugular access sites can provide superior control, allowing one snare to stabilize the fragment while the other achieves capture [14]. In our case, initial attempts via femoral access alone were unsuccessful due to the fragment's mobility within the cardiac chambers and suboptimal capture angle. The addition of jugular access allowed for coordinated manipulation, repositioning the fragment from the right ventricle into the right atrium with favorable orientation. The final successful retrieval was achieved using a larger 10-mm snare from the femoral approach, highlighting the importance of having multiple device sizes available and the willingness to employ multiaccess strategies when initial attempts fail. The use of prophylactic anticoagulation during the retrieval procedure remains controversial, with practices varying among institutions. In our case, subcutaneous heparin was administered to reduce the risk of thrombus formation on the foreign body surface and potential thromboembolic complications during manipulation. However, this must be balanced against bleeding risks, particularly if vascular injury occurs during retrieval. Surgical extraction via sternotomy or thoracotomy is reserved for cases where endovascular retrieval fails or is not feasible, such as fragments firmly embedded in the myocardium, fragments with associated large thrombi, or situations where endovascular manipulation poses excessive risk [15]. Given the invasiveness and morbidity of surgical approaches, every effort should be made to achieve percutaneous retrieval.

Prevention of catheter fracture and embolization requires a multifaceted approach encompassing proper insertion technique, patient selection, catheter material selection, surveillance protocols, and prompt recognition of warning signs. The lateral approach to subclavian vein cannulation, with the puncture site lateral to the mid-clavicular line, significantly reduces the risk of pinch-off syndrome by avoiding the costoclavicular space [10]. Alternatively, internal jugular vein insertion eliminates pinch-off risk entirely and has become increasingly preferred in many institutions. When subclavian access is necessary, ultrasound guidance and anatomic landmark awareness are essential. One of the most important yet frequently overlooked preventive measures is obtaining chest radiography before elective port removal, particularly for catheters that have been in place for extended periods (>12 months) or in patients with symptoms suggestive of catheter dysfunction [11]. This simple measure can identify catheter fracture before attempted removal, allowing for controlled retrieval planning rather than emergent intervention. Patients should be counseled to report any new symptoms such as chest pain, palpitations, positional flow difficulties during port access, or inability to flush the port, as these may indicate catheter complications [12]. For long-term catheters, periodic chest radiography (e.g., annually) may be considered to assess catheter integrity, particularly in high-risk patients with subclavian insertion or prolonged dwell times [1,2]. Newer catheter materials and designs with improved resistance to compression and fracture are continually being developed, and catheter selection should consider the anticipated duration of use and patient-specific factors [3].

This case report, while illustrative of the successful management of a rare complication, has inherent limitations. As a single case, it cannot provide statistical evidence regarding optimal retrieval techniques or comparative outcomes. The patient's asymptomatic presentation may not reflect the full spectrum of clinical scenarios encountered with catheter embolization. Additionally, long-term follow-up beyond three months was not available to assess for any delayed complications. Future research should focus on large-scale registry data to better define risk factors for catheter fracture, optimal surveillance strategies, comparative effectiveness of different retrieval techniques, and long-term outcomes following foreign body retrieval. Cost-effectiveness analyses of routine pre-removal imaging versus management of complications could inform clinical practice guidelines.

## Conclusions

This case highlights the critical importance of pre-removal radiologic evaluation for long-term central venous catheters, particularly those with subclavian insertion. Most catheter fractures present asymptomatically, making intraoperative vigilance essential; any shortened or irregular catheter tip warrants immediate imaging. Endovascular retrieval achieves >90% success rates when performed promptly with appropriate multiaccess strategies. Prevention through optimal insertion techniques (lateral subclavian or jugular approaches), patient education, and clinical awareness can reduce the risk of this rare but potentially catastrophic complication.
